# Improving compliance to serum calcium and urate testing guidelines for patients with renal stones: a Two-Cycle audit

**DOI:** 10.51894/001c.143427

**Published:** 2025-08-26

**Authors:** Obinna Enemoh, Mayowa Adefehinti, Uchizi Mvalo, Obichukwu Iwunna

**Affiliations:** 1 Department of Urology Chesterfield Royal Hospital, Chesterfield UK.; 2 Department of Urology Peterborough City Hospital, Peterborough UK; 3 Department of Acute Medicine Sherwood Forest Hospitals NHS Foundation. GP Training, Sherwood Forest Program, NHSE East Midlands UK

**Keywords:** Clinical audit, quality improvement, renal stones, calculi, calcium, Urology

## Abstract

**INTRODUCTION:**

Renal stones develop when urinary solutes crystallize into solid deposits within the urinary tract. The 2019 National Institute for Health and Care Excellence (NICE) and British Association of Urological Surgeons (BAUS) guidelines recommend serum calcium and urate testing for all patients with renal or ureteric stones. This single-center audit aimed to assess compliance with these guidelines in a local urology department and implement quality improvement interventions to enhance adherence.

**METHODS:**

We conducted a retrospective two-cycle audit on patients admitted with renal stones by the urology team at Chesterfield Royal Hospital, United Kingdom. Patient information and admission investigations were reviewed using the hospital’s electronic medical records. Data were obtained from electronic medical records and assessed for compliance with calcium and urate testing guidelines. Interventions included educational sessions for clinicians, reminder posters, and updates to admission documentation. Pre- and post-intervention results were compared using statistical analysis.

**RESULTS:**

A total of 70 patients were included (36 in the first cycle; 34 in the second). In the first cycle, urate testing was performed in 2 patients (5.6%), while calcium testing was performed in 31 (86.1%). Following the intervention, urate testing increased to 13 patients (38.2%; p = 0.00087) and calcium testing reached 100% compliance (p=0.0241).

**CONCLUSION:**

This audit identified poor adherence to urate and calcium testing guidelines for patients with renal stones. Educational and process interventions significantly improved compliance, achieving 100% for calcium testing and a more than sixfold increase in urate testing. Sustained improvement will require continued quality improvement measures.

## INTRODUCTION

Renal stones form when chemicals in concentrated urine crystallize into solid deposits within the urinary tract.^1^ The condition may be asymptomatic or present with painful colic or hematuria, is more common in males than in females,^2^ and can lead to serious complications, including kidney failure. Renal stones can occur secondary to metabolic syndrome, genetic disorders, endocrinopathies, or drugs, hence necessitating a multidisciplinary approach to management^3^ and a thorough evaluation within prescribed guidelines. There are various types of renal stones including calcium-containing, uric acid, ammonium, infection stones, which put affected individuals at risk of recurrent stone formation.^4^

The 2019 National Institute for Health and Care Excellence (NICE) recommends measuring serum calcium levels and considering stone analysis in adults with renal or ureteric stones.^5^ The British Association of Urological Surgeons (BAUS) renal colic 2019 guidelines likewise, recommend both calcium testing and urate testing for all patients with renal stones.^6^ Calcium testing is an inexpensive approach that can detect treatable underlying conditions that are responsible for renal stones, such as primary hyperparathyroidism. Similarly, elevated uric acid may indicate other conditions that could potentially be treated through medical management or lifestyle modifications.

This study is a single-center retrospective observational audit at a National Health Service (NHS) Trust hospital in the United Kingdom (UK). Using a quality improvement methodology, we seek to evaluate our current testing practices for calcium and urate against the standards of care recommended for patients with renal colic. This study reinforces the importance of continuous quality improvement projects in clinical care that emphasizes the necessity of routine assessment to ensure adherence to standards of care.

## METHODS

### Aims

The primary objective was to enhance adherence to evidence-based recommendations for calcium and urate testing in patients with renal colic at the NHS Trust Hospital, aiming to increase compliance from baseline by a minimum of 50% within 6 months.

### Ethical Considerations

Our study was registered and approved by the Clinical Quality Improvement Audit Unit at the Chesterfield Royal Hospital. Ethical approval was not required as this was a clinical audit. Data collection and analysis followed the hospital’s data privacy policy, and we excluded audit opt-outs. Patient details were anonymized, and no patient identifiable data was shared.

### Study area

The study was conducted on patients admitted by the Urology Department at Chesterfield Royal Hospital (CRH), United Kingdom.

### Study design

Our quality improvement project used a Plan-Do-Study-Act (PDSA) methodology^7^ alongside a retrospective two-cycle audit to assess adherence with serum calcium and urate testing in accordance with the 2019 NICE and BAUS guidelines for patients admitted with renal stones. The corresponding recommendations on testing for patients with renal stones are shown below (Table 1).

**Table 1.** Testing guidelines for adult patients with renal stones

**Testing Guidelines for Renal Stones**	**Recommendations**
National Institute for Health and Care Excellence (NICE)- (GG118, 2019)	Measure serum calcium for adults with ureteric or renal stones. *Rationale for calcium testing: to identify hypercalcemia, an indication of a treatable condition such as primary hyperthyroidism that is responsible for recurrent calcium stones.*
British Association of Urological Surgeons (BAUS)- (2019)	Investigations should include serum calcium and urate in all patients with suspected renal colic or stones. *Rationale for urate testing: to identify risk for uric acid and calcium oxalate stones crystallization.*

Source:

NICE (2019). Renal and ureteric stones: assessment and management. NICE guideline NG118, 1.7.2. BAUS (2019). Diagnostic Pathways in Suspected Renal Colic

Although NICE and BAUS have not published the recommended institutional compliance targets for calcium and urate testing, our study aimed for complete adherence (100%) with these testing guidelines. The first audit cycle was carried out between August and September 2024 and collected baseline data on calcium and urate testing among eligible patients. We conducted a problem root cause analysis using a fishbone diagram to identify causes for non-adherence with NICE and BAUS guidelines and then implemented interventions to improve adherence. The intervention program was implemented over a twelve-week period between October and December 2024. The initial intervention consisted of sending our findings as an audit update alert via email to all clinicians on the department’s mailing list. Our findings were also summarized in an educational poster (Appendix 1), which was displayed in different clinical areas including all acute surgical wards, the surgical assessment unit, and the various physicians’ offices. The findings were also presented at the Urology Department’s bi-monthly quality improvement meeting. During this meeting, we discussed strategies for improving resident physicians’ knowledge as well as recommendations for other process changes, and secured approval for project plans implementation. Subsequently, educational sessions were organized for the resident physicians and nursing staff who work closely with the urology team. The educational sessions were conducted in November and December of 2024. The presentations were delivered by one of the authors (OE) and under the supervision of a consultant urologist.

After the educational intervention period, we carried out a second audit cycle on patients admitted with renal colic from January to February 2025 to assess improvement in urate and calcium testing. We presented the findings at the Urology Department’s bi-monthly quality improvement meeting in April 2025, with recommendations for long-term process changes. The PDSA method for this study is outlined in the table below (Table 2).

**Table 2.** PDSA methodology for audit

**PDSA**	**First PDSA Cycle (First Audit)**	**Second PDSA Cycle (Second Audit)**
Plan	Collect baseline data to check the current compliance with calcium and urate testing guidelines for patients with renal stones. Get coding data for patients who meet the inclusion criteria.	Plan an educational intervention to resident physicians and nursing staff to enhance their adherence with calcium and urate testing for patients with renal stones. Plan data collection to evaluate adherence rates with guidelines.
Do	Collect retrospective data over an eight-week period. Analyze the collected data.	Implement the educational intervention and collect data after the intervention and begin analysis.
Study	Compare data to expected outcomes. Conduct a fishbone analysis to identify causes for the problem.	Complete data analysis and evaluate the findings against baseline data and the aim statement.
Act	Adopt the plan for change: educating the resident physicians and nursing staff about the guidelines. Plan for the second PDSA cycle.	Evaluate the findings and standardize the improvement. Continue the improvement efforts to sustain gains.

### Inclusion and exclusion criteria

All patients, aged 16 years old and older, who were admitted through the emergency department to the surgical assessment unit (SAU) with a confirmed diagnosis of renal colic, were included in the study. Patients under the age of 16 or those who were not confirmed to have renal colic were excluded.

### Data Collection

In collaboration with the information technology (IT) department at the hospital, we extracted data for patients with a discharge ICD code of renal colic and excluded audit opt-outs. Renal stone diagnosis was confirmed by a CT scan, which was also verified by one of the authors (UM) for inclusion. Patients’ information was reviewed on the hospital electronic medical records and data collected using a Google Form (Google, Mountain View, CA). All extracted data was analyzed by the first author using Microsoft Excel (Microsoft Corp., Redmond, WA, US) with the help of a statistician (see acknowledgements). We compared adherence rates for calcium and urate testing before and after our interventions. A z-statistics was used to analyze sample proportions to get confidence intervals and a z-test to determine statistical significance.

## RESULTS

**Figure 1.**
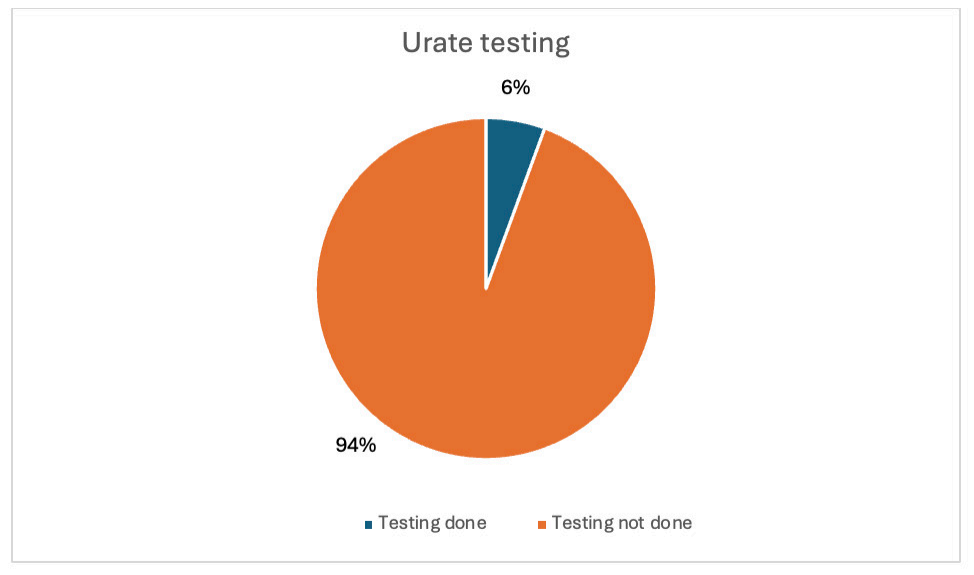
Results for first cycle urate testing

**Figure 2.**
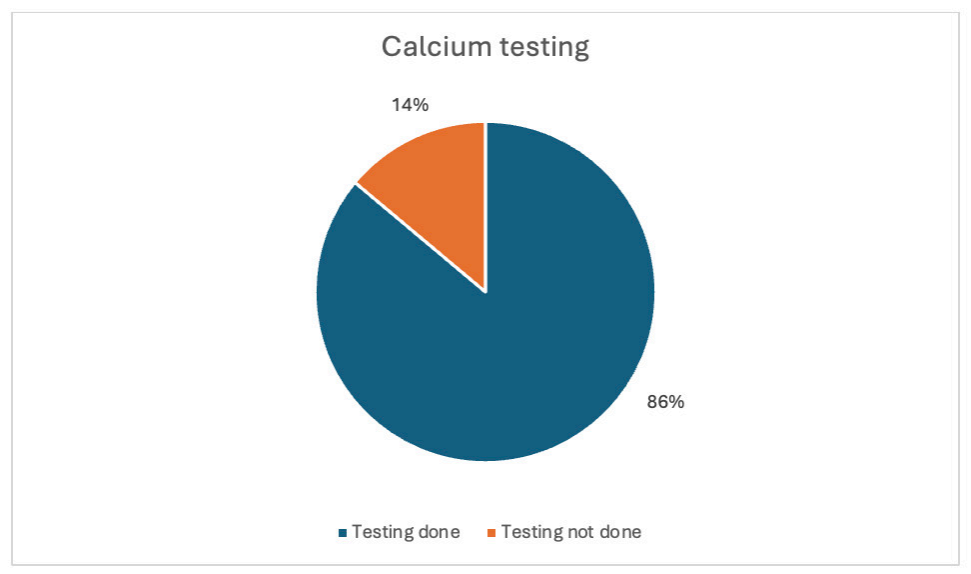
Results for first cycle calcium testing

A total of 70 patients met the criteria and were included across both audit cycles. The average age of patients was 56.78 years (range 17-95). In the first audit cycle, thirty-six (36) eligible patients were included and studied. Of these 36 patients, only two participants (5.6%, 95% CI 0.0 – 13.0%) had serum urate testing in that admission (Figure 1). On the other hand, 31 out of 36 eligible patients (86.1%, 95% CI 74.8–97.4%) had calcium testing on admission. (Figure 2). For this cycle, most of the patients did not have urate tested while calcium testing was relatively higher.

After the intervention period, we conducted a second cycle audit and included 34 participants. Thirteen participants (38.2%, 95% CI 21.9–54.6%) had urate testing on admission, which represents a substantial increase from the 5.6% observed from our first cycle (p = 0.00087). For calcium testing, all 34 patients in this audit cycle (100%, 95% CI 100–100%) had serum calcium level measured, showing a significant improvement from the previous cycle (p=0.0241). A comparison of the results from the two audit cycles is in Table 3.

**Table 3.** Comparisons of testing between first and second cycle audit comparisons

**Investigation**	**First PDSA Cycle (n=36)**	**Second PDSA Cycle (n=34)**	**p-value**
Urate testing	2 (5.6%; 95% CI 0.0–13.0%)	13 (38.2%; 95% CI 21.9–54.6%)	0.00087
Calcium testing	31 (86.1%; 95% CI 74.8–97.4%)	34 (100%; 95% CI 100–100%)	0.0241

## DISCUSSION

Renal stones are a common urological condition and a frequent cause of acute hospital presentation, mainly due to painful colic symptoms. In the UK, it accounted for around 90,000 hospital admissions between 2019- 2020.^8^ Renal stone management has a significant economic impact on the National Health Service (NHS). According to a UK study, the average cost per episode was between £1277 and £2887. In 2010, the estimated cost of managing renal stones was between £190 million and £324 million in England alone. These costs included imaging, inpatient stays with or without surgical intervention, and outpatient clinic appointments.^9^

Calcium stones make up about 80% of all renal stones while uric acid stones comprise 8-10% of all renal stones.^10,11^ These urine substances contribute to significant renal stones complications; hence, early detection is important to identify and treat underlying and reversible causes of renal stones. Primary hyperparathyroidism can be present in patients with recurrent calcium-containing renal stones secondary to hypercalcemia (increased intestinal calcium reabsorption resulting in elevated calcium in the serum) and hypercalciuria (increased calcium excretion in the urine). This predisposes affected individuals to symptomatic stone formation which can be reversed through surgical excision.^12^ Hyperuricemia, another etiologic factor in renal stone formation, can be influenced by diet, genetics, and certain medical conditions like metabolic syndrome, obesity, diabetes mellitus, and hypertension. Conservative strategies such as hydration, urine alkalization, or xanthine oxidase inhibitors are used to manage these stones and prevent their recurrence.^13^ Despite the effectiveness of these therapeutic modalities, preventive strategies remain more impactful in reducing the risk of recurrence, physical, and financial burdens.^14^ As a result, NICE and BAUS have put together these testing guidelines for all patients to identify these conditions and institute appropriate management.

A Plan-Do-Study-Act (PDSA) quality improvement methodology was used to evaluate our current practice and institute interventions for improvement. The peak age for developing renal stones is between 40-60 years for men.^15^ Based on our study, the average age of surveyed patients was 56.78 years, which is comparable to data from available literature. From the first PDSA cycle, our audit showed that urate and calcium testing were at 5.6% and 86.1% respectively. This did not meet the recommended standards of investigation for patients with renal colic. Calcium testing was relatively higher because it is included as part of the routine liver function tests (LFTs) done for most admitted surgical patients at CRH. The admission paper booklet in the hospital (used for clerking patients) contains a list of blood tests, which includes calcium but excludes uric acid. Therefore, this serves as a reminder to clinicians to request calcium blood test.

Clinician awareness of quality standards and required baseline investigations for newly diagnosed patients with renal stones were the main drivers for change. Urology admissions at CRH are done by tier 2 clinicians (Foundation Year 2 physicians, Core Surgical Trainees, General Practice trainees and Clinical Fellows) before getting reviewed by Urology Specialists. Since these second tier physicians regularly rotate through different specialties and have varied backgrounds, their knowledge and experience of urology guidelines and standards vary. We identified a need for education on appropriate management guidelines of patients with renal stones; therefore, we implemented an intervention program to improve the physicians’ knowledge on these standards. The intervention consisted of teaching sessions for those posted to the surgical assessment unit, placing posters in key clinical areas and offices, and holding weekly departmental teaching and discussion sessions around change implementation. Our findings were also shared with all clinicians via email and presented to the bimonthly urology audit meeting. In addition to these interventions, we updated the list of investigations to be done on admission or prior to discharge to include calcium and urate and amended our procedure to have the results reviewed by the Urology team either on the ward or in follow-up clinics. After implementing these changes, we conducted a reaudit in the second PDSA cycle, which showed an improvement in urate and calcium testing. The initial urate testing rate of 5.6% improved to 38.2%, while calcium testing improved from 86.1% to 100%. Despite these positive findings, there remains room for improvement, especially for urate measurement.

In the long term, we plan to modify the paper admission booklet to include a box for writing the calcium and urate results, which will serve as a reminder for clinicians to order these tests when admitting patients. Another measure which we recommend is the introduction of electronic order sets for renal stone investigations. The implementation of standard electronic order sets has been shown to reduce variation in physician investigations and improve health care efficiency.^16^ As this is a continuous improvement project, these measures will be reviewed at different time intervals to ensure standards are met and maintained. We suggest a yearly audit to check compliance, sustain any gains from these cycles, and keep improving.

### Limitations

Our study is limited by the short duration of the PDSA cycles and the small data sample size. Moreover, our study was conducted in a single medical center with a sample size of 70 over two audit cycles, which may not accurately reflect long term changes in the practice or generalizability of the findings to other settings. Reliance on electronic records and a brief post-intervention window was another limitation which prevented the assessment whether the improvements in the resident physicians’ and the nursing staff knowledge of guidelines can be attributed to the sustained adherence to guidelines over time without the need for periodic educational reinforcement.

## CONCLUSION

Renal stones are a common acute urology presentation and cause significant morbidity. The 2019 NICE and BAUS guidelines recommend testing serum calcium and urate levels as part of investigating patients with renal stones. This audit assessed our compliance to these testing guidelines. From our first cycle, we obtained baseline compliance data, which identified inadequate urate and calcium testing. Following intervention, a re-audit showed improvement, achieving over 50% increased adherence in both tests. To maintain adherence to testing guidelines, we need to implement continuous quality improvement interventions to support sustained improvements in patient care.

### Conflict of Interest

None.
